# In Vitro Effects of PRP, Ozonized PRP, Hyaluronic Acid, Paracetamol, and Polyacrylamide on Equine Synovial Fluid-Derived Mesenchymal Stem Cells

**DOI:** 10.3390/life15101558

**Published:** 2025-10-04

**Authors:** Denisa Bungărdean, Emoke Pall, Zsofia Daradics, Maria Popescu, Mirela Alexandra Tripon, Alexandru Florin Lupșan, Cristian Mihăiță Crecan, Ianu Adrian Morar, Alexandru Nicolescu, Florin Dumitru Bora, Ioan Marcus

**Affiliations:** 1 Department of Pathophysiology, Faculty of Veterinary Medicine, University of Agricultural Sciences and Veterinary Medicine (UASVM) Cluj-Napoca, Mănăştur Street 3–5, 400372 Cluj-Napoca, Romania; denisa.bungardean@usamvcluj.ro (D.B.); ioan.marcus@usamvcluj.ro (I.M.); 2 Department of Infectious Diseases, Faculty of Veterinary Medicine, University of Agricultural Sciences and Veterinary Medicine Cluj-Napoca, Calea Mănăştur nr. 3–5, 400372 Cluj-Napoca, Romania; emoke.pall@usamvcluj.ro; 3 Department of Internal Medicine, Faculty of Veterinary Medicine, University of Agricultural Sciences and Veterinary Medicine (UASVM) Cluj-Napoca, Mănăştur Street 3–5, 400372 Cluj-Napoca, Romania; sofia.daradics@usamvcluj.ro; 4 Equine Clinic, Faculty of Veterinary Medicine, University of Agricultural Sciences and Veterinary Medicine (UASVM) Cluj-Napoca, Mănăştur Street 3–5, 400372 Cluj-Napoca, Romania; maria.popescu@usamvcluj.ro; 5 Department of Reproduction, Obstetrics and Veterinary Gynecology, Faculty of Veterinary Medicine, University of Agricultural Sciences and Veterinary Medicine (UASVM) Cluj-Napoca, Mănăştur Street 3–5, 400372 Cluj-Napoca, Romania; mirela.tripon@usamvcluj.ro (M.A.T.); iancu.morar@usamvcluj.ro (I.A.M.); 6 Department of Anesthesiology and Surgery, University of Agricultural Sciences and Veterinary Medicine (UASVM) Cluj-Napoca, Mănăştur Street 3–5, 400372 Cluj-Napoca, Romania; alexandru-florin.lupsan@usamvcluj.ro (A.F.L.); cristian.crecan@usamvcluj.ro (C.M.C.); 7 Laboratory of Chromatography, Advanced Horticultural Research Institute of Transylvania, Faculty of Horticulture and Business for Rural Development, University of Agricultural Sciences and Veterinary Medicine, 400372 Cluj-Napoca, Romania; 8 Viticulture and Oenology Department, Advanced Horticultural Research Institute of Transylvania, Faculty of Horticulture and Business in Rural Development, University of Agricultural Sciences and Veterinary Medicine (UASVM) Cluj-Napoca, Mănăştur Street 3–5, 400372 Cluj-Napoca, Romania

**Keywords:** equine therapy, horse, mesenchymal stem cells, musculoskeletal issues, orthopedy, platelet-rich plasma, regenerative medicine

## Abstract

Musculoskeletal disorders are a major cause of lameness in horses, often necessitating innovative regenerative strategies to restore joint function and improve quality of life. This study investigated the effects of platelet-rich plasma (PRP), ozonized PRP, hyaluronic acid, paracetamol, and polyacrylamide hydrogel (NOLTREX^®^) on the behavior of mesenchymal stem cells (MSCs) derived from equine synovial fluid. Synovial fluid samples were collected under strict cytological criteria to ensure viability, followed by in vitro expansion and phenotypic characterization of MSCs. Cultures were supplemented with the tested preparations, and cellular proliferation and viability were evaluated at 24 h, 72 h, and 7 days. PRP significantly promoted MSC proliferation in a time- and dose-dependent manner, with maximal effect at 10%. Hyaluronic acid stimulated growth, most pronounced at 1 mg/mL, while paracetamol induced a concentration-dependent proliferative response, strongest at 100 μg/mL. NOLTREX displayed a biphasic effect, initially inhibitory at high concentrations but stimulatory at 7 days. Ozonized PRP showed concentration-dependent redox activity, with lower doses maintaining viability and higher doses producing an initial suppression followed by delayed stimulation. Collectively, these findings support the therapeutic potential of PRP and related biologic preparations as intra-articular regenerative therapies in equine medicine, while underscoring the importance of dose optimization and standardized protocols to facilitate clinical translation.

## 1. Introduction

Lameness due to musculoskeletal disease is the most common diagnosis in equine veterinary care and a leading cause of morbidity and loss of performance in athletic horses [1,2].

Regenerative medicine comprises therapies that restore the function of cells and tissues impaired by injury, aging, or disease, with the goal of reestablishing normal architecture and function [1]. Platelet-rich plasma (PRP) has emerged as a regenerative alternative to intra-articular corticosteroids, delivering growth factors that support tissue repair, cartilage regeneration, and the management of musculoskeletal disorders [3,4]. First used in humans for osteoarthritis and cartilage lesions [5,6], PRP is now applied intra-articularly in equine practice [7]. In horses, PRP has shown promising results in reducing pain, controlling inflammation, and improving joint function [3,8,9]. The regenerative potential of PRP derives mainly from its platelet-derived growth factors [7]. These factors promote chondrocyte proliferation and differentiation [3]. Over the past two decades, PRP has been widely used for musculoskeletal disorders, showing benefits in pain relief, function, and tissue repair, though issues of standardization and long-term efficacy persist [10]. Other autologous biologics, including stromal vascular fraction (SVF) and autologous conditioned serum (ACS), have also gained attention for their regenerative and anti-inflammatory properties in osteoarthritis [11].

Synovial fluid is a valuable source of mesenchymal stem cells (MSCs), whose numbers increase during joint inflammation [12]. Equine models closely mimic human joint disease and are widely used to evaluate MSC-based therapies [13]. MSCs can be derived from bone marrow, adipose tissue, umbilical cord, and synovial fluid, and are recognized for their regenerative and immunomodulatory properties [14]. PRP further enhances the proliferation of synovial fluid-derived MSCs and has shown clinical benefits in osteoarthritis [15,16].

The clinical use of MSCs in equine cartilage repair remains limited by factors such as cell source, donor variability, immune response, and implantation strategies [17]. These challenges highlight the need for adjuvants to improve MSC survival and chondrogenic potential.

Few studies have examined the regenerative potential of PRP combined with ozone, although intra-articular ozone therapy is considered safe in equine osteoarthritis [18,19,20]. Both autologous and allogeneic MSCs can also be administered intra-articularly without major adverse effects, supporting their clinical use [21]. Practical factors such as tissue harvesting, culture conditions, and delivery methods remain critical for MSC viability and therapeutic outcomes [22].

A recent review emphasized the lack of studies comparing intra-articular agents on MSC biology, despite their growing use in equine orthopedics [23]. Hyaluronic acid (HA) is valued for its viscoelastic and chondroprotective effects, but its influence on MSC proliferation and stemness remains unclear. Polyacrylamide hydrogels (e.g., Noltrex^®^) are used as joint fillers, yet cellular data are limited. Paracetamol, though common as an analgesic, has rarely been studied for possible redox-mediated effects on MSCs. PRP combined with ozone is an especially novel but scarcely investigated approach, while mitotherapy has recently shown safety in equine osteoarthritis [24]. Closing these gaps is key to defining the optimal microenvironment for MSC-based therapies [23].

In horses with osteochondritis dissecans treated by arthroscopy, intra-articular PRP or HA failed to improve outcomes and increased synovial effusion, raising doubts about their efficacy [25]. This underscores the need for controlled studies to clarify their cellular effects [25].

This study aimed to isolate and characterize MSCs from equine synovial fluid and assess their responses to regenerative preparations, focusing on proliferative and survival capacity under supplementation with PRP, ozonized PRP, hyaluronic acid (HA), polyacrylamide hydrogel (Noltrex^®^), and paracetamol. Prior evidence suggests that PRP and ozonized PRP stimulate MSC proliferation, HA supports a favorable microenvironment, and polyacrylamide and paracetamol elicit dose-dependent effects. These findings underscore the translational potential of MSC–biologic combinations in equine regenerative medicine and the need for optimized intra-articular protocols.

## 2. Materials and Methods

### 2.1. Examination and Selection of Horses

#### 2.1.1. Experimental Animals

The study was conducted at the Equine Clinic, Faculty of Veterinary Medicine, University of Agricultural Sciences and Veterinary Medicine of Cluj-Napoca, Romania. A total of 42 horses were screened, of which 27 (aged 2–17 years) met the inclusion criteria (absence of systemic disease, localized orthopedic condition, suitability for synovial fluid collection) and were enrolled. Horses with joint pathology or altered synovial fluid (arthritis, septic arthritis, arthrosis, traumatic joint injuries, osteochondritis dissecans, fractures) were excluded. All procedures complied with EU Directive 2010/63/EU and national legislation and were approved by the institutional Ethics Committee (523/30 July 2025); informed owner consent was obtained. Synovial fluid was collected only from clinically healthy joints. The cohort consisted mainly of sport horse breeds with some light draft horses. Animal characteristics are summarized in Supplementary Table S1. The inclusion of both sexes, multiple breeds, and a broad age range improved representativeness. A sample size of *n* = 24 provided sufficient statistical power (Cohen’s d > 0.7, α = 0.05, power = 0.8), with low within-group variability (RSD% < 2%), supporting robust interpretation of outcomes.

#### 2.1.2. Clinical and Orthopedic Examination

All horses underwent full clinical and orthopedic evaluation, including health status, lameness examination, and gait analysis. Flexion tests, imaging (radiography/ultrasonography), and peri-neural anesthesia were applied when indicated. Synovial fluid was aspirated only from joints free of pathology on clinical and radiographic assessment. Joints with prior injections, visible lesions, or insufficient fluid were excluded. Sampling was performed mainly from radiocarpal, tarsocrural, or femoropatellar joints, which yield sufficient synovial fluid with minimal risk.

### 2.2. Collection and Analysis of Synovial Fluid

#### 2.2.1. Synovial Fluid Collection and Processing

Synovial fluid was aseptically collected by arthrocentesis under general anesthesia for MSC isolation [26]. Horses were premedicated with xylazine (1.1 mg/kg IV), induced with ketamine (2.2 mg/kg IV), intubated, and maintained on isoflurane in oxygen with continuous monitoring. The puncture site was aseptically prepared, local anesthesia was provided with lidocaine, and arthrocentesis was performed using a 21 G needle. Between 3–15 mL synovial fluid was aspirated, mainly from stifle, carpal, hock, or coxofemoral joints, depending on accessibility. Samples were collected into sterile syringes and EDTA tubes for subsequent analyses.

#### 2.2.2. Synovial Fluid Analysis

Synovial fluid was assessed macroscopically for color and viscosity, with normal clear, pale yellow, and viscous fluid used as reference. Samples with blood contamination or abnormal discoloration were excluded [27]. Microscopic evaluation included total nucleated cell counts, neutrophil percentage, and screening for erythrocytes or bacterial contamination, using standard cytological staining [27,28]. All samples were anonymized and analyzed by a blinded independent examiner.

### 2.3. Mesenchymal Stem Cell Isolation and Morphological Evaluation

Synovial fluid was transported at 4–8 °C and processed within 4 h. After centrifugation, the cell pellet was resuspended in propagation medium and cultured under standard conditions for MSC isolation. Cell adhesion was monitored at 24, 48, and 72 h; non-adherent cells were removed after 72 h, and the medium was changed every 48 h. Cultures showing signs of contamination were excluded [29]. Adherent cell morphology was assessed microscopically, and cells were passaged at 70% confluence. MSCs were characterized by immunophenotypic and functional analyses [30], then cryopreserved in DMSO-containing medium and stored in liquid nitrogen until use.

#### Flow Cytometry Immunophenotyping

Synovial fluid-derived cells (passages 2–3) were detached, washed, and incubated with fluorochrome-conjugated antibodies against CD90, CD44, and CD105 (positive markers), and CD34 and CD45 (negative markers), alongside isotype controls. Samples were analyzed by flow cytometry, gating on viable singlet cells. Antibody selection was based on previous reports validating the equine MSC immunophenotype [29,30].

### 2.4. Preparation of Platelet-Enriched Plasma and Ozonized PRP

Platelet-rich plasma (PRP) was prepared with the Arthrex ACP^®^ system following established protocols [31,32,33,34]. After centrifugation, the platelet-enriched fraction was collected and used according to the manufacturer’s instructions. Ozonized PRP was obtained by exposing PRP to medical ozone at 5, 10, and 75 µg/mL using a standard clinical generator, with mixing performed through a closed system as previously described [18,19]. The preparations were diluted to the required concentrations and applied to MSC cultures.

### 2.5. Determination of the Effects of Regenerative Preparations on Stem Cell Cultures

#### 2.5.1. Supplementation of Stem Cells with Regenerative Preparations

To assess regenerative effects, synovial fluid-derived MSCs were treated with PRP (5–10%), ozonized PRP (5–75 µg/mL), hyaluronic acid (0.25–1.0 mg/mL), paracetamol (50–100 µg/mL), or polyacrylamide hydrogel (Noltrex^®^, 1–4%). Detailed concentrations are provided in Supplementary Table S2. Cultures (1 × 10^5^ cells/well) were evaluated for proliferation and cytotoxicity at 24 h, 72 h, and 7 days. Preparations were coded, and assays were performed by blinded investigators.

#### 2.5.2. Determination of Stem Cells Viability

Cell viability was assessed with the CCK-8 assay, which measures the metabolic reduction in WST-8 to formazan. Absorbance was read at 450 nm after 4 h incubation, and viability was expressed as a percentage of untreated controls. Measurements were performed at 24 h, 72 h, and 7 days, in triplicate, and analyzed by treatment type and concentration (Supplementary Figure S1) [35].

### 2.6. Statistical Assessment

All experiments were performed in triplicate, and data are presented as mean ± SD. Reproducibility was confirmed by RSD < 2%. Differences between treatments were analyzed by one-way ANOVA with Duncan’s post hoc test, with significance set at *p* < 0.05. Correlations were evaluated with Pearson’s coefficient, and graphics were generated in R and GraphPad Prism (version 10.6). Power analysis based on pilot data indicated that the total sample size (*n* = 24 donor horses) provided 80% power to detect moderate-to-large effects at α = 0.05, considering donor variability as the main source of variance. Independent biological replicates derived from these donors were distributed across all treatment groups. Technical replicates minimized measurement error but were not counted as independent biological units.

## 3. Results

### 3.1. Cytological Analysis of Synovial Fluid

Synovial fluid samples were screened macroscopically and microscopically. Only samples with normal clarity, viscosity, and minimal contamination were used, while those with erythrocytes, >10% neutrophils, or bacterial presence were excluded [36,37,38,39,40]. This cytological screening ensured preservation of MSC viability and functional potential.

Cytological criteria were applied before MSC isolation. Normal synovial fluid is acellular or mildly cellular, with few neutrophils and no erythrocytes. The presence of red blood cells suggests trauma or contamination, while >10% neutrophils indicates inflammatory or septic conditions [31].

Altered synovial fluid composition not only serves as a diagnostic marker but also directly affects cell culture. Blood contamination and elevated neutrophils release inflammatory mediators and enzymes that impair MSC viability, adhesion, and differentiation. Although MSCs can modulate neutrophil activity, their regenerative potential is reduced under highly inflammatory conditions [37,38,40,41,42].

**Figure 1:**
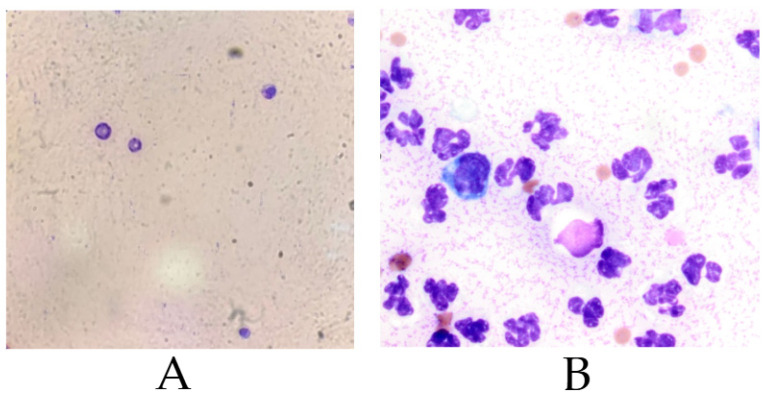
Representative cytological features of equine synovial fluid samples used for MSCs evaluation. (**A**) Non-inflammatory profile, suitable for cell culture; (**B**) Inflamed and contaminated profile, indicative of septic synovitis. Bright-field microscopy, magnification 400× (objective 40×).

In septic or osteoarthritic joints, synovial fluid often shows increased IL-1β/IL-1Ra, turbidity, and reduced viscosity, changes that justify exclusion from culture [36,43,44,45]. PRP and hyaluronic acid can modulate these effects, improving MSC culture success [7,25]. Accordingly, samples with inflammatory or contaminated profiles (Figure 1B) were excluded, while those with low cellularity and no contamination (Figure 1A) were used. This strict selection preserved experimental consistency and stem cell functionality [40,46].

High neutrophil density indicates inflammatory or septic conditions (e.g., synovitis, infectious arthritis) and creates an unfavorable environment for MSC survival through reactive by-products and cytokines such as IL-1β [25,42,43,44,45]. Therefore, only samples with low inflammation and no microbial or blood contamination (profile as in Figure 1A) were used, while those resembling Figure 1B were excluded.

### 3.2. Morphology of Isolated Mesenchymal Stem Cells

**Figure 2:**
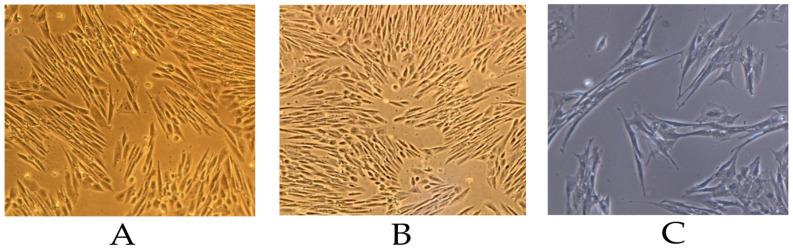
Microscopic evaluation of mesenchymal stem cells isolated from equine synovial fluid at 50–70% confluence. (**A**) 60% confluence; (**B**) 70% confluence; (**C**) cell clusters. Phase-contrast microscopy, magnification 200× (objective 20×).

Following cytological screening, MSC cultures showed optimal expansion. Morphology was assessed by phase-contrast microscopy at 50–70% confluence to confirm phenotypic stability and proliferative behavior (Figure 2) [41].

#### Immunophenotypic Characterization

Flow cytometry confirmed the MSC phenotype, with high expression of CD90, CD44, and CD105 (>90%) and negligible CD34/CD45 (<3%), consistent with ISCT criteria (Supplementary Figures S1 and S2). Together with successful trilineage differentiation, these data validate the MSC identity of the isolated synovial fluid-derived cells.

Equine synovial fluid-derived MSCs showed the expected spindle-shaped, fibroblast-like morphology with elongated cytoplasm, oval nuclei, and absence of degenerative features. Cultures maintained morphological stability from 50% to 70% confluence, with alignment into parallel arrays at higher density but no evidence of senescence or spontaneous differentiation. Occasional small clusters retained typical morphology. Overall, the cultures remained viable and stable, supporting their suitability for proliferation and downstream assays [41].

### 3.3. Effects of Regenerative Preparations on Stem Cell Cultures

MSCs cultured with PRP showed concentration-dependent differences in viability, with 10% PRP consistently producing the strongest and most sustained increase, while 5–7% maintained values close to baseline with only minor fluctuations. MSCs exposed to sodium hyaluronate showed dose- and time-dependent effects, with 1 mg/mL producing the strongest increase in viability at 72 h and 7 days, while lower concentrations remained close to baseline. Paracetamol produced modest, concentration-dependent effects, with 50 μg/mL slightly reducing MSC viability, while 100 μg/mL-maintained values comparable to controls across all time points. Noltrex^®^ showed concentration-dependent effects, with 4% increasing MSC viability above controls, while 1–2% produced values largely comparable or slightly lower. Ozonized PRP produced variable effects on MSC viability: 5% maintained values near controls, 7.5% caused moderate reductions, and 10% led to a stronger initial decrease followed by partial recovery by day 7.

#### 3.3.1. Effect of PRP

**Figure 3:**
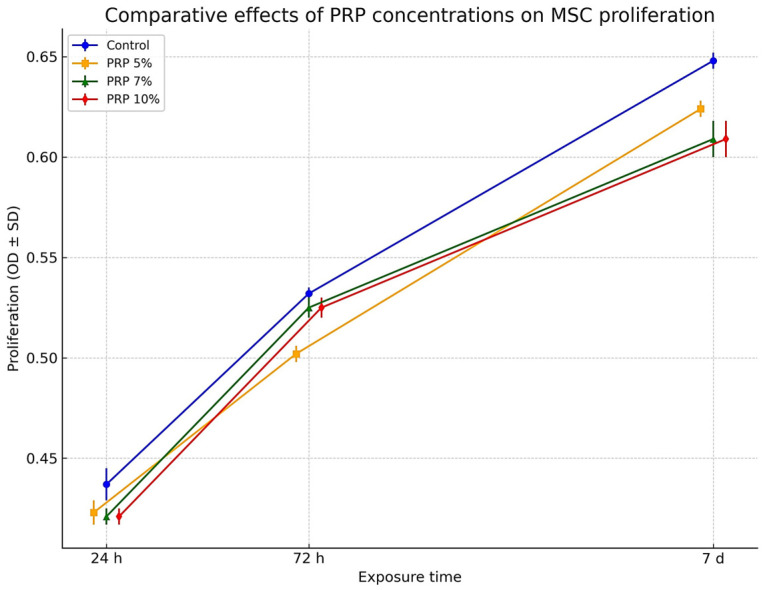
Comparative effect of PRP dilutions (5%, 7%, 10%) versus control on stem cell proliferation across different exposure times (24 h, 72 h, 7 days). Values are expressed as mean ± SD of three replicates. Error bars indicate standard deviation. Figure 3 denote statistically significant differences (*p* < 0.05, Duncan’s test).

**Figure 4:**
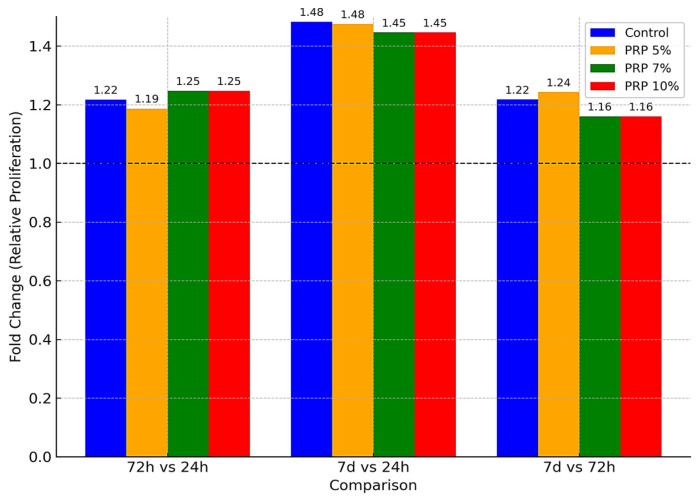
Comparative fold change in stem cell proliferation following PRP treatment at concentrations of 5%, 7%, and 10% versus control across different exposure times (72 h vs. 24 h, 7 d vs. 24 h, and 7 d vs. 72 h). Data are expressed as relative fold change (mean ± SD) from three independent replicates, calculated relative to the baseline at 24 h.

The effect of PRP on MSC proliferation was both time- and dose-dependent, with all tested concentrations supporting cell growth compared to untreated controls (Figure 3 and Figure 4, Table 1). At lower doses (5% and 7%), proliferation remained close to baseline at 24 h, followed by a modest but consistent increase at 72 h and a more pronounced stimulation after 7 days. In contrast, 10% PRP produced a sustained proliferative response across all time points, clearly outperforming the lower concentrations at later stages. Figure 3 illustrates the temporal progression of optical density values, showing that all groups followed a similar upward trajectory, yet diverged progressively over time, with 10% PRP maintaining the highest proliferation throughout the culture period. Complementary fold-change analysis (Figure 4) demonstrates that the strongest proliferative gain occurred during the first 72 h, followed by a plateau phase in which differences between concentrations became more evident. These findings indicate that PRP enhances MSC proliferation in a dose-responsive manner, with the 10% concentration providing the most consistent and robust stimulation, particularly during the later phase of culture.

Overall, statistical analysis confirmed highly significant effects of both PRP concentration and exposure time on MSCs proliferation (F = 1080–1407; *p* < 0.001). The progressive assignment of superscript letters (a → b → c) validated temporal differences within each concentration, while fold-change analysis emphasized the marked proliferative increase between baseline and long-term exposure. The strongest proliferative effect was consistently observed at 10% PRP after 7 days, which represented the peak response across all experimental conditions.

#### 3.3.2. Effect of Sodium Hyaluronate

MSCs were treated with a commercial HA formulation (Hyalgan, 20 mg/2 mL) at final concentrations of 1, 0.5, and 0.25 mg/mL (Table 2). All experiments were performed in triplicate, with reproducibility confirmed by low variability (RSD% < 2%). At 1 mg/mL, HA induced the most pronounced temporal separation in MSC proliferation, with minimal activity at 24 h, a marked increase by 72 h, and the highest response observed at 7 days.

Statistical analysis confirmed a strong time-dependent effect, with each interval clearly distinguished from the others. The intermediate concentration (0.5 mg/mL) showed a similar temporal pattern, though with a lower overall magnitude. Proliferation increased significantly between 24 h, 72 h, and 7 days, with statistical analysis confirming clear separation of time points and consistent reproducibility across replicates. At the lowest concentration (0.25 mg/mL), MSC proliferation was relatively higher at baseline but followed the same time-dependent increase, peaking at 7 days. Statistical analysis confirmed significant differences between time points, with this group showing the lowest variability and excellent reproducibility. Comparative analysis showed that all HA concentrations followed a parallel time-dependent increase, with proliferation highest at 1 mg/mL, intermediate at 0.5 mg/mL, and lowest at 0.25 mg/mL. The strongest overall effect was observed at 1 mg/mL after 7 days. Across all HA conditions, data reproducibility was confirmed by low variability, while statistical analysis demonstrated significant effects of both concentration and exposure time on MSC proliferation.

**Figure 5:**
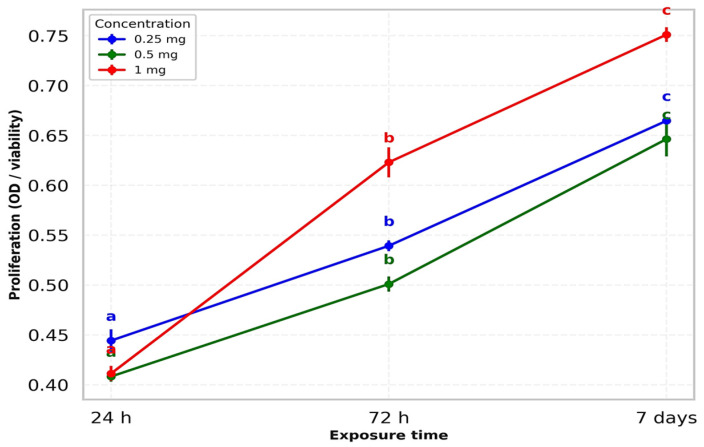
Interaction between hyaluronic acid concentration and exposure time on stem cell proliferation (mean ± SD, one-way ANOVA with Duncan’s test). Data are expressed as mean ± SD from three independent replicates. Different letters (a–c) indicate statistically significant differences (*p* < 0.05).

**Figure 6:**
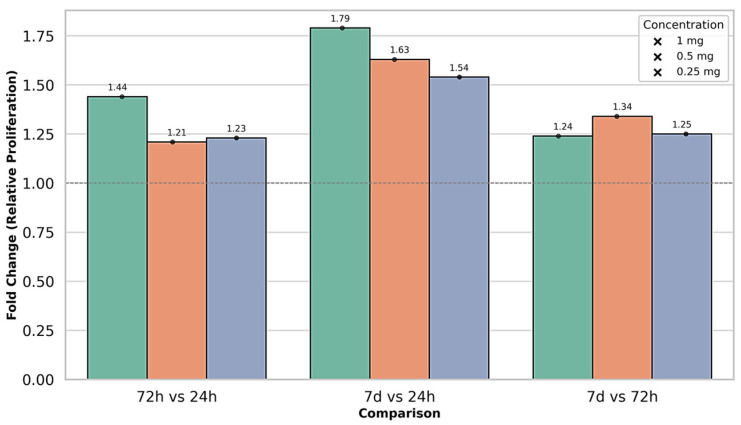
Comparative fold change in stem cell proliferation following hyaluronic acid treatment at different concentrations and exposure times (mean ± SD, relative to baseline at 24 h).

Figure 5 illustrates the interaction between HA concentration and exposure time, confirming a clear dose- and time-dependent proliferative effect. At 24 h, differences between concentrations were minimal, but by 72 h distinct stratification emerged, with 1 mg/mL showing the highest stimulation. This effect was further amplified at 7 days, when 1 mg/mL consistently produced the strongest proliferative response compared to the lower doses. Figure 5 illustrates the parallel temporal progression of all HA concentrations, with the steepest increase observed at 1 mg/mL, confirming its stronger proliferative effect. Narrow error bars support high reproducibility, while the statistical separation of time points validates the dose- and time-dependent pattern, in line with the results summarized in Table 2. Fold-change analysis relative to the 24 h baseline (Figure 6) demonstrated both early stimulation at 72 h and sustained long-term enhancement at 7 days, with all values consistently above 1.0. The strongest effect was observed at 1 mg/mL, confirming a graded, concentration-dependent response. These results complement the statistical findings in Table 2 and the temporal trends illustrated in Figure 5.

#### 3.3.3. Effect of Paracetamol

**Figure 7:**
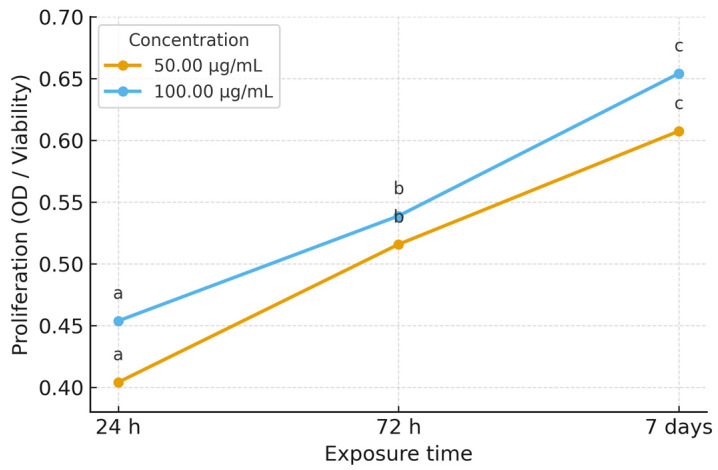
Comparative effect of paracetamol (50 μg/mL and 100 μg/mL) on stem cell proliferation at different exposure times (24 h, 72 h, 7 days). Values are expressed as mean ± SD of three replicates. Error bars indicate standard deviation. Different letters (a–c) indicate statistically significant differences (*p* < 0.05). Statistical differences (*p* < 0.05) were determined using one-way ANOVA with Duncan’s post hoc test.

**Figure 8:**
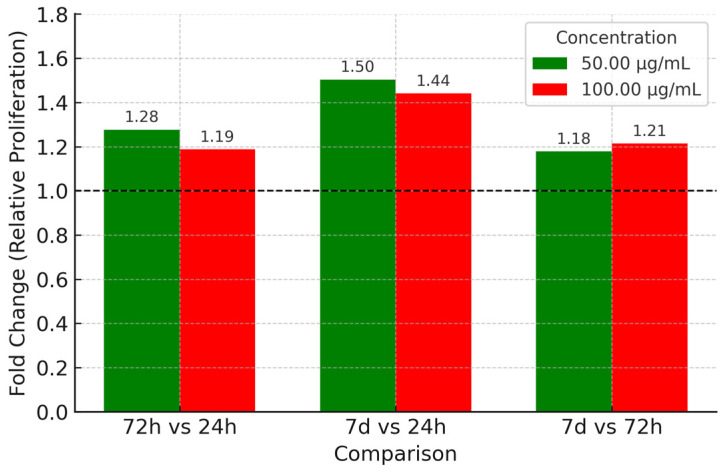
Comparative fold change in stem cell proliferation following Paracetamol treatment at concentrations of 50 μg/mL and 100 μg/mL across different exposure times (72 h vs. 24 h, 7 d vs. 24 h, and 7 d vs. 72 h). Data are expressed as relative fold change (mean ± SD) from three independent replicates, calculated relative to the baseline at 24 h.

Table 3 summarizes MSC proliferation under paracetamol exposure. Both concentrations (50 and 100 μg/mL) showed a time-dependent increase, with significant rises at 72 h and 7 days. The proliferative response was slightly stronger at 100 μg/mL compared to 50 μg/mL. Statistical analysis confirmed significant effects of both concentration and exposure time, with high reproducibility across replicates. At 50 μg/mL, proliferation increased progressively from 24 h to 7 days, while 100 μg/mL produced a slightly stronger response following the same temporal pattern. Figure 7 illustrates these trends, with clear separation between time points confirmed by post hoc testing. Fold-change analysis relative to baseline (Figure 8) showed modest stimulation under paracetamol exposure, with increases evident at both 72 h and 7 days. The effect was slightly greater at 50 μg/mL than at 100 μg/mL.

When comparing 7 days to 72 h, fold-change values indicated a modest additional increase, suggesting that the stimulatory effect persisted but at a slower rate than in the early phase. Overall, these findings corroborate the absolute values in Table 3 and the trends in Figure 7, confirming a consistent dose- and time-dependent increase in MSC proliferation, with the strongest response after 7 days.

#### 3.3.4. Effect of Polyacrylamide Gel

Table 4 summarizes the proliferation of MSCs cultured with NOLTREX^®^ (polyacrylamide gel, 4%), with all assays performed in triplicate and showing low variability (RSD% < 2%). In the control group, proliferation increased gradually over time, confirming the normal baseline progression. At 1%, NOLTREX followed a comparable pattern, with slightly higher values at later stages, suggesting a mild stimulatory effect. At 10%, proliferation was markedly reduced at 24 h but showed a steady recovery by 72 h and 7 days, indicating that the initial inhibitory effect was transient. At 4%, values were moderately reduced at 24 h but increased progressively thereafter, reaching levels significantly higher by the end of the culture period. Statistical testing confirmed that, across all concentrations, temporal differences were significant, with the strongest proliferative responses consistently recorded after 7 days. These results demonstrate that NOLTREX^®^ exerts dose- and time-dependent effects, with low concentrations supporting proliferation, while higher concentrations initially inhibit cell growth before allowing recovery and stimulation.

**Figure 9:**
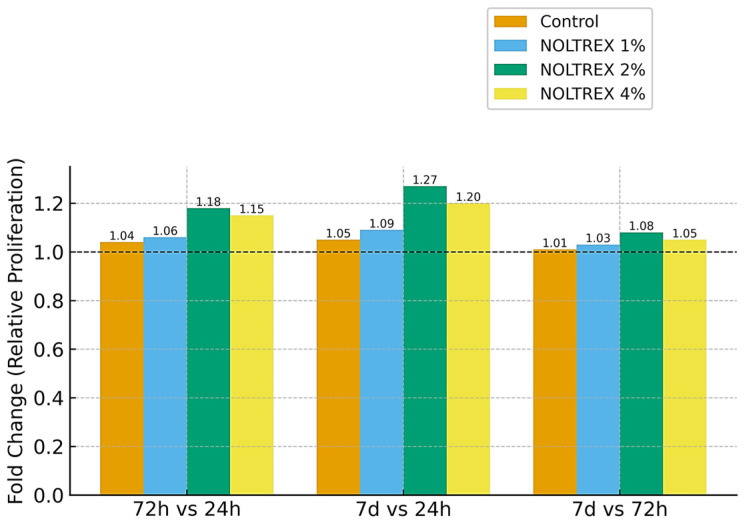
Comparative fold change in stem cell proliferation following NOLTREX (polyacrylamide gel, 4%) treatment at dilutions of 1%, 2%, and 4% versus control across different exposure times (72 h vs. 24 h, 7 d vs. 24 h, and 7 d vs. 72 h). Data are expressed as relative fold change (mean ± SD) from three independent replicates, calculated relative to the baseline at 24 h.

**Figure 10:**
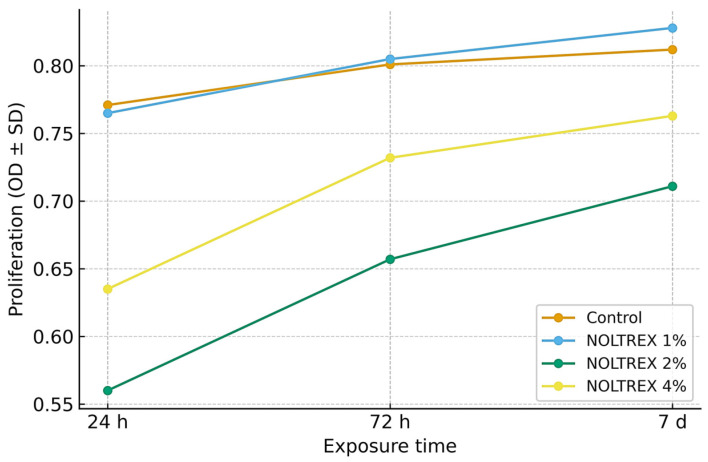
Comparative effect of NOLTREX (polyacrylamide gel, 4%) dilutions (1%, 2%, 4%) versus control on cell proliferation across different exposure times (24 h, 72 h, 7 days). Values are expressed as mean ± SD of three replicates. Error bars indicate standard deviation.

The one-way ANOVA results support these interpretations, with F-values ranging from 8.9 to 23.5 and *p*-values between 0.001 and 0.017, confirming the statistical significance of both concentration and time as determinants of cellular response. Duncan’s test further validated the temporal progression within each concentration, consistently stratifying exposure times into distinct statistical groups. Across all concentrations tested, NOLTREX modulated proliferation in a time- and dose-dependent manner (Table 4). At 1%, proliferation values remained comparable to control at all time points. At 2%, values were reduced at 24 h (≈0.55–0.60 OD; group “a”), but increased progressively to 0.65–0.67 at 72 h (group “b”) and 0.70–0.71 at 7 days (group “c”). At 4%, proliferation was moderately reduced at 24 h (≈0.61–0.66 OD; group “a”), followed by increases to 0.71–0.75 at 72 h (group “b”) and 0.73–0.78 at 7 days (group “c”). The highest proliferation values were consistently observed at 4% after 7 days (Figure 9 and Figure 10).

The graphical representation (Figure 9 and Figure 10) shows the interaction between NOLTREX concentration (1%, 2%, 4%) and exposure time on MSC proliferation, confirming the dose- and time-dependent pattern reported in Table 4. At 24 h, values ranged from 0.55–0.77 OD, with only minor differences between groups (Duncan’s “a”). By 72 h, stratification became evident, as the 2% and 4% groups rose to ≈0.65–0.73 OD, while control and 1% reached ≈0.80 OD. At 7 days, proliferation peaked in the 4% group (≈0.77 OD), followed by 2% (≈0.71 OD), with control and 1% maintaining slightly higher values (≈0.81–0.83 OD). Narrow error bars confirmed high reproducibility (RSD% < 2%). Overall, NOLTREX induced progressive increases over time, with the strongest stimulation at 4% after 7 days.

The fold-change analysis (Figure 9) illustrates proliferation relative to the 24 h baseline. Between 72 h and 24 h, increases were modest (1.04–1.18), with 1% and 4% NOLTREX showing slightly stronger effects. By 7 days, fold changes rose to 1.05–1.27, confirming a dose-dependent enhancement with prolonged exposure. Comparisons between 7 days and 72 h showed only moderate gains (1.01–1.08), indicating that most stimulation occurred within the first 72 h, followed by a plateau. Across all conditions, fold-change values remained >1.0, confirming that NOLTREX consistently promoted MSC proliferation.

Taken together, Figure 9 and Figure 10 show that NOLTREX stimulates stem cell proliferation in a dose- and time-dependent manner, with the highest concentration (4%) yielding the most robust long-term effect. The combination of progressive statistical separation (a → b → c), elevated fold changes, and low variability across replicates supports the strong and reproducible proliferative potential of NOLTREX treatment.

#### 3.3.5. Effect of Ozonized PRP

Table 5 shows that ozonized PRP influenced MSC proliferation in a clear concentration- and time-dependent manner. Control cultures displayed a gradual increase from 0.770 ± 0.005 at 24 h to 0.811 ± 0.003 at 7 days. At 5 µg/mL, proliferation paralleled control, with values rising from ≈0.791 to ≈0.841, suggesting preserved basal activity with a mild stimulatory effect. At 10 µg/mL, proliferation was markedly suppressed at 24 h (0.525–0.595 OD) but recovered to 0.708–0.713 by day 7, indicating a biphasic response. At 75 µg/mL, early inhibition was less pronounced (0.607–0.662 OD at 24 h), followed by the strongest rebound, peaking at 0.782 by day 7. Statistical testing confirmed significant temporal differences (Duncan’s test, a → b → c) and ANOVA supported the effects of both dose and time (F = 8.9–23.6; *p* = 0.001–0.017). Overall, low-dose ozonation maintained proliferation, intermediate doses induced transient inhibition, and high doses promoted the strongest long-term enhancement, underscoring ozone concentration as a key modulator of PRP’s regenerative effects.

**Figure 11:**
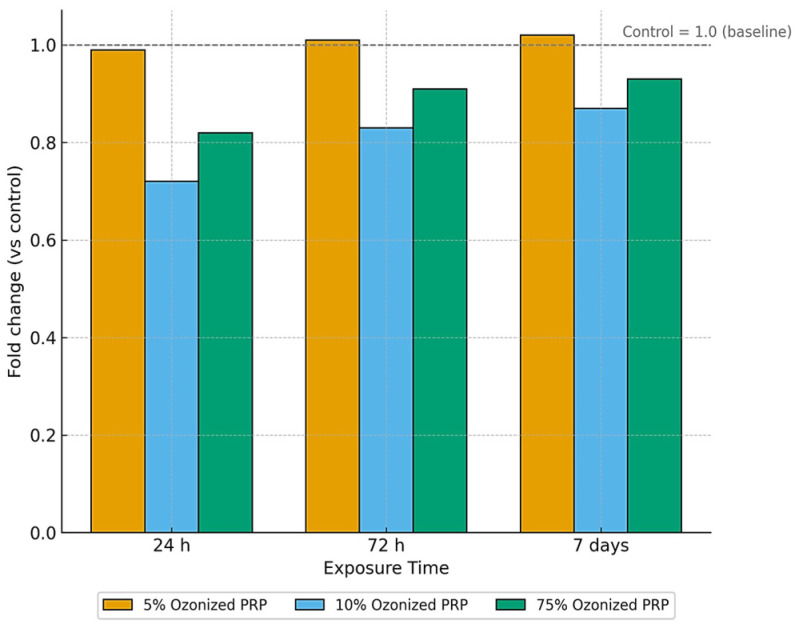
Fold change in MSC proliferation relative to control after exposure to ozonized PRP (5, 10, 75 µg/mL) at 24 h, 72 h, and 7 days.

Fold-change analysis relative to untreated controls (Figure 11) supported these patterns. At 5 µg/mL, proliferation remained essentially unchanged across all time points (≈1.0–1.02). At 10 µg/mL, a pronounced early suppression was observed (≈0.72 at 24 h), followed by partial recovery at 72 h (≈0.83) and 7 days (≈0.87). In contrast, at 75 µg/mL, proliferation showed moderate inhibition at 24 h (≈0.82), but a robust rebound by day 7 (≈0.92–0.94). Together, these data confirm that ozonized PRP exerts a transient, dose-dependent suppressive effect, with higher concentrations ultimately driving the strongest long-term recovery.

## 4. Discussion

PRP enhanced MSC proliferation in a clear time- and dose-dependent manner, with the strongest stimulation observed at 10% after 7 days. These findings reinforce the regenerative potential of MSCs for equine cartilage repair, although clinical translation remains constrained by variability in cell source, donor-related factors, and implantation protocols [17]. This pattern is consistent with previous studies describing PRP as a rich source of platelet-derived growth factors (PDGF, TGF-β, VEGF) and cytokines, which are known to modulate the cell cycle, adhesion, and metabolic activity of MSCs [47]. The immunophenotypic profile (CD90^+^, CD44^+^, CD105^+^, CD34^−^, CD45^−^) together with trilineage differentiation confirmed that the isolated cells met ISCT criteria for MSCs, ensuring that the observed responses reflect bona fide MSC behavior with translational relevance.

A likely mechanism is the activation of proliferative pathways such as PI3K/Akt and MAPK, triggered by growth factors [48]. The similar responses at moderate and high concentrations suggest a proliferative ‘ceiling,’ beyond which no further enhancement occurs.

These results emphasize the role of PRP dosage in modulating MSC responses. Lower concentrations sustain viability with limited stimulation, whereas higher levels (≈10%) consistently enhance proliferation. Clinically, this supports PRP as a regenerative adjunct in MSC-based therapies, with the equine model providing a relevant translational platform. While literature reports encouraging outcomes for PRP in musculoskeletal disorders, reproducibility remains limited by heterogeneity in preparations, variable platelet concentrations, and lack of standardized protocols. Similar challenges are noted for other autologous biologics such as SVF and ACS, where reported benefits are offset by inconsistent methodologies and outcomes.

To facilitate comparability, we summarize the biological profile of PRP obtained with the Arthrex ACP^®^ system (Supplementary Table S4), which consistently yields leukocyte-poor PRP with reproducible platelet enrichment and elevated growth factors, supporting the plausibility of the proliferative effects observed. Nonetheless, clinical outcomes remain variable: in horses with osteochondritis dissecans, intra-articular PRP was linked to increased effusion and poorer flexion test results [25], raising concerns about its therapeutic value in some contexts. More recent studies indicate that combining chondroprogenitors (CPCs) with PRP yields superior hyaline-like cartilage regeneration compared to MSCs alone [23], suggesting that PRP stimulation may reproduce part of CPCs’ benefits and highlighting the importance of the microenvironment in stem cell efficacy.

HA exposure induced a clear dose- and time-dependent increase in MSC proliferation, with the strongest effect at 1 mg/mL after 7 days. This aligns with previous evidence identifying HA as a bioactive matrix component that engages receptors such as CD44 and RHAMM to activate proliferative pathways and protect against oxidative stress [49]. The stronger response at higher concentrations suggests a ligand density-dependent effect, while HA’s supportive microenvironment may also help preserve stemness. These findings are consistent with the broader role of equine MSCs in modulating inflammation and promoting musculoskeletal repair [14].

Translationally, these findings are relevant as HA is already widely used in articular and regenerative therapies, and its combination with MSCs may enhance efficacy. However, further studies are required to confirm whether the in vitro proliferative effects are maintained in vivo and whether they influence differentiation or genomic stability. Supporting this, animal studies have shown that CPCs combined with HA reduce OARSI scores and synovial inflammation in osteoarthritis models [23]. Our data suggest that MSC–HA interactions may share similar trophic mechanisms, reinforcing their potential for equine orthopedic and regenerative applications [23].

Paracetamol exposure produced a sustained, dose-dependent increase in MSC proliferation, with stronger effects at 100 μg/mL. Beyond its known analgesic and antipyretic role in equine musculoskeletal pain [50], emerging evidence indicates that paracetamol can modulate MSC gene expression, including COL10A1 and other chondrogenic markers [51].

A plausible mechanism involves moderate ROS generation [52], which may activate antioxidant pathways and adaptive responses that enhance proliferation [53]. The stronger effect at higher concentrations likely reflects transient redox-mediated activation rather than cytotoxicity, while the non-linear trend suggests that lower doses may disrupt metabolic enzymes, whereas higher doses trigger compensatory mechanisms sustaining viability.

Despite its proliferative effects, the use of paracetamol in regenerative contexts requires caution, as its known hepatotoxicity raises concerns about genomic stability and differentiation potential of MSCs. Reports of anti-proliferative and cytotoxic effects via caspase- and JNK/p38-mediated pathways further underline these risks [54]. Given the contradictory literature, additional studies are needed to determine whether the observed response is transient or therapeutically relevant. Interestingly, the adaptive redox effect resembles CPC preconditioning strategies, suggesting that paracetamol may indirectly modulate the MSC microenvironment and enhance resistance to oxidative stress [23].

NOLTREX^®^ hydrogel showed a biphasic, concentration-dependent effect on MSC proliferation. At low concentrations (5%), proliferation was similar to controls, while higher doses (10% and 75%) caused transient inhibition at 24 h, followed by recovery and significant stimulation at 7 days. This adaptive response may reflect temporary effects on nutrient diffusion or cell–matrix signaling, after which the hydrogel environment supports proliferation. Such biphasic behavior aligns with previous reports on polyacrylamide and other scaffolds, highlighting the importance of biomaterial context in regenerative strategies. Clinically, these findings suggest potential benefits of NOLTREX^®^, though further studies are needed to balance its stimulatory effects with possible stress responses. Ozonized PRP induced distinct and concentration-dependent effects on MSCs proliferation. At 5%, proliferation was comparable to control throughout the culture period, suggesting that mild ozonation did not impair basal activity and may even provide a slight stimulatory effect. At higher concentrations (10% and 75%), proliferation was initially suppressed at 24 h but recovered progressively, with 75% showing the strongest stimulation by 7 days.

This response pattern suggests a redox-dependent mechanism. Ozonation generates ROS, which at moderate levels can function as signaling molecules that enhance proliferation and metabolic activity. At higher concentrations, the transient inhibitory effect likely reflects oxidative stress, followed by adaptive activation of endogenous antioxidant defenses. Such biphasic behavior is consistent with the concept of oxidative preconditioning, whereby controlled stress primes cells for increased resilience. Nevertheless, not all biological therapies trigger such transient inflammatory responses. For instance, intra-articular mitotherapy in horses was well tolerated and did not induce clinically perceptible inflammation, highlighting its potential as a safe alternative biologic approach [24].

Compared with standard PRP, ozonized PRP exerted a stronger long-term stimulatory effect, particularly at 75%, suggesting an enhanced potential for MSC expansion. Nevertheless, prior to clinical translation, further studies are needed to determine whether these adaptive responses influence genomic stability, differentiation capacity, or long-term functionality of MSCs. Recent studies reported that CPCs performed better than MSCs, but remained comparable or even inferior to chondrocytes [23]. Our findings suggest that ozonized PRP may ‘empower’ MSCs to acquire a functional profile closer to that of CPCs.

Overall, our results show that PRP and ozonized PRP had the strongest proliferative effects on MSCs, followed by HA, NOLTREX^®^, and paracetamol. These findings highlight the role of growth factors, HA, and redox balance in creating a supportive microenvironment and support the development of MSC-based therapies in equine joint disease. However, further in vivo studies are needed to address limitations such as immunogenicity and genomic stability.

Our results should also be interpreted in light of existing safety data, as intra-articular administration of both autologous and allogeneic MSCs has been reported to elicit only mild, self-limiting inflammation in healthy horses, without systemic adverse effects [21]. These issues are consistent with previously highlighted gaps regarding the clinical use of MSCs in equine medicine, where factors such as cell source, passage number, and administration protocols remain incompletely standardized [22]. These issues mirror broader challenges reported for equine MSCs applications, where reproducibility is affected by donor variability, immune responses, and the need for optimized implantation strategies [17]. Future directions should include direct MSCs–CPC comparisons under identical conditions, as well as clinical testing of MSCs + ozonized PRP/HA combinations in equine models.

While the present study provides novel insights into the effects of PRP, ozonized PRP, HA, paracetamol, and polyacrylamide (Noltrex^®^) on equine synovial fluid-derived MSCs, several aspects warrant consideration. First, the experiments were performed under controlled in vitro conditions, which enabled precise assessment of cellular responses but did not fully reproduce the complex mechanical and inflammatory environment of the equine joint. Second, although MSCs were derived from multiple donor horses to capture inter-individual variability, this variability also introduces heterogeneity that may influence specific outcomes. Third, the study focused on individual treatments, and future work could explore combined or sequential therapeutic strategies that may better reflect clinical practice. Finally, the number of biological replicates was sufficient to detect moderate-to-large effects, but larger-scale studies may further refine the observed trends. Another important limitation is the absence of direct characterization of the PRP preparations employed. Although we relied on the Arthrex ACP^®^ system, which is reported to yield leukocyte-poor PRP with reproducible enrichment of platelets and growth factors, we did not measure platelet counts, cytokine composition, or growth factor concentrations in the preparations used. This omission limits our ability to correlate specific cellular responses with defined PRP components. Future studies should therefore prioritize the biochemical and cellular characterization of PRP batches, as this would improve reproducibility, enable cross-study comparisons, and support the establishment of standardized protocols. Taken together, these considerations do not detract from the robustness of our findings; rather, they highlight directions for future research aimed at translating these results into clinical applications.

## 5. Conclusions

This study demonstrated that regenerative preparations such as PRP, hyaluronic acid, paracetamol, NOLTREX, and ozonized PRP exert distinct dose- and time-dependent effects on MSCs derived from equine synovial fluid. PRP (10%) and hyaluronic acid (1 mg/mL) showed the most consistent proliferative stimulation, confirming their established role as intra-articular biologic therapies. Ozonized PRP exhibited a biphasic effect, with low concentrations maintaining viability and high concentrations inducing transient inhibition followed by recovery, suggestive of oxidative preconditioning. NOLTREX demonstrated delayed but significant stimulation at higher concentrations, while paracetamol unexpectedly enhanced proliferation at therapeutic doses, indicating a potential modulatory role in redox balance. Collectively, these findings consolidate existing knowledge on intra-articular biologics and highlight their potential to support MSC proliferation and survival in regenerative equine medicine. At the same time, our data confirm the preservation of MSC morphology and fundamental characteristics in vitro, yet the broader immunomodulatory and differentiation potential of MSCs warrants further investigation. Future studies should therefore explore the long-term implications of these therapies on MSC differentiation, genomic stability, and in vivo regenerative performance, while establishing standardized protocols to ensure reproducible clinical outcomes.

## Figures and Tables

**Table 1:** Comparative effects of PRP concentrations (5%, 7%, 10%) and exposure times on stem cell proliferation and metabolic activity (mean ± SD, RSD%, ANOVA, Duncan’s test).

Concentration	Exposure Time	Mean ± SD (Replicate 1)	RSD%	Mean ± SD (Replicate 2)	RSD%	Mean ± SD (Replicate 3)	RSD%	F-Value	*p*-Value
Control	24 h	0.430 ± 0.008 i	1.873	0.435 ± 0.008 i	1.873	0.446 ± 0.008 i	1.873	5.54	0.043
Control	72 h	0.530 ± 0.003 e	0.497	0.531 ± 0.003 e	0.497	0.535 ± 0.003 e	0.497	0.43	0.668
Control	7 d	0.645 ± 0.004 a	0.556	0.647 ± 0.004 a	0.556	0.652 ± 0.004 a	0.556	3.67	0.091
PRP 5%	24 h	0.418 ± 0.006 j	1.316	0.422 ± 0.006 j	1.316	0.429 ± 0.006 j	1.316	5.05	0.052
PRP 5%	72 h	0.499 ± 0.004 h	0.718	0.501 ± 0.004 h	0.718	0.506 ± 0.004 h	0.718	2.21	0.191
PRP 5%	7 d	0.620 ± 0.004 b	0.578	0.625 ± 0.004 b	0.578	0.627 ± 0.004 b	0.578	6.13	0.036
PRP 7%	24 h	0.418 ± 0.004 k	0.856	0.420 ± 0.004 k	0.856	0.425 ± 0.004 k	0.856	11.42	0.009
PRP 7%	72 h	0.520 ± 0.005 f	0.959	0.530 ± 0.005 f	0.959	0.524 ± 0.005 f	0.959	2.96	0.127
PRP 7%	7 d	0.600 ± 0.009 c	1.403	0.610 ± 0.009 c	1.403	0.617 ± 0.009 c	1.403	7.84	0.021
PRP 10%	24 h	0.418 ± 0.004 l	0.856	0.420 ± 0.004 l	0.856	0.425 ± 0.004 l	0.856	3.39	0.103
PRP 10%	72 h	0.520 ± 0.005 g	0.959	0.530 ± 0.005 g	0.959	0.524 ± 0.005 g	0.959	4.84	0.056
PRP 10%	7 d	0.600 ± 0.009 d	1.403	0.610 ± 0.009 d	1.403	0.617 ± 0.009 d	1.403	9.76	0.013

Values are expressed as mean ± SD of three replicates, with RSD% (Relative Standard Deviation) indicating measurement precision; values below 2% reflect high reproducibility. Different superscript letters (a–l) denote statistically significant differences between exposure times within the same concentration, according to Duncan’s multiple range test (*p* < 0.05). The F-values represent the test statistic from one-way ANOVA, where higher values indicate stronger between-group differences relative to within-group variance, while the calculated *p*-values (ranging from 0.009 to 0.668) indicate that some treatment doses and exposure times have significant effects, whereas others do not reach statistical significance. Comparisons are relative to the 24 h baseline, which reflects the initial proliferation response. Overall, the data indicate that PRP stimulates stem cell proliferation in a dose- and time-dependent manner, with the most pronounced effect observed at 10% after 7 days.

**Table 2:** Comparative effects of hyaluronic acid concentrations and exposure times on stem cell proliferation and metabolic activity (mean ± SD, RSD%, ANOVA, Duncan’s test).

Concentration (mg)	Exposure Time	Mean ± SD (Replicate 1)	RSD%	Mean ± SD (Replicate 2)	RSD%	Mean ± SD (Replicate 3)	RSD%	F-Value	*p*-Value
Control	24 h	0.412 ± 0.006 a	1.3	0.412 ± 0.006 a	1.3	0.412 ± 0.006 a	1.3	F = 9.58	*p* = 0.002
	72 h	0.624 ± 0.004 b	0.6	0.624 ± 0.004 b	0.6	0.624 ± 0.004 b	0.6	F = 11.50	*p* = 0.001
	7 days	0.750 ± 0.007 c	0.9	0.750 ± 0.007 c	0.9	0.750 ± 0.007 c	0.9	F = 23.43	*p* = 0.001
1 mg	24 h	0.421 ± 0.003 a	0.713	0.407 ± 0.002 a	0.491	0.406 ± 0.003 a	0.739	F = 18.95	*p* = 0.002
	72 h	0.607 ± 0.002 b	0.329	0.621 ± 0.003 b	0.483	0.641 ± 0.003 b	0.468	F = 22.54	*p* = 0.001
	7 days	0.754 ± 0.004 c	0.478	0.743 ± 0.004 c	0.485	0.756 ± 0.007 c	0.867	F = 19.73	*p* = 0.002
0.5 mg	24 h	0.411 ± 0.003 a	0.730	0.407 ± 0.007 a	1.611	0.407 ± 0.007 a	1.611	F = 7.21	*p* = 0.025
	72 h	0.497 ± 0.002 b	0.402	0.497 ± 0.002 b	0.402	0.509 ± 0.009 b	1.679	F = 6.58	*p* = 0.030
	7 days	0.668 ± 0.007 c	1.080	0.637 ± 0.008 c	1.285	0.634 ± 0.005 c	0.723	F = 12.97	*p* = 0.007
0.25 mg	24 h	0.432 ± 0.003 a	0.694	0.445 ± 0.006 a	1.251	0.456 ± 0.007 a	1.438	F = 18.36	*p* = 0.002
	72 h	0.533 ± 0.004 b	0.676	0.542 ± 0.003 b	0.488	0.543 ± 0.003 b	0.552	F = 21.65	*p* = 0.001
	7 days	0.664 ± 0.002 c	0.301	0.665 ± 0.005 c	0.752	0.665 ± 0.005 c	0.752	F = 16.74	*p* = 0.003

The control for HA confirmed the reproducibility and consistency of the obtained results. MSC proliferation in the untreated group was 0.412 ± 0.006 (RSD% = 1.3) at 24 h, increased to 0.624 ± 0.004 (RSD% = 0.6) at 72 h, and reached 0.750 ± 0.007 (RSD% = 0.9) at 7 days. Statistical analysis demonstrated significant differences between exposure times (F = 9.58–23.43, *p* = 0.001–0.002). At the intermediate concentration of 0.5 mg/mL, proliferation was 0.409 ± 0.004 (RSD% = 1.1) at 24 h, 0.503 ± 0.005 (RSD% = 1.0) at 72 h, and 0.652 ± 0.006 (RSD% = 0.9) at 7 days, with ANOVA confirming significant time-dependent differences (*p* < 0.01). At the lowest concentration (0.25 mg/mL), values were 0.444 ± 0.005 (RSD% = 1.2) at 24 h, 0.538 ± 0.003 (RSD% = 0.6) at 72 h, and 0.664 ± 0.004 (RSD% = 0.7) at 7 days, again showing statistically significant differences across time points. For all tested concentrations, ANOVA results and consistently low RSD values (<2%) confirmed the reproducibility and robustness of the findings. Values are expressed as mean ± SD of three replicates, with RSD% (Relative Standard Deviation) indicating measurement precision; values below 2% reflect high reproducibility. Different superscript letters (a–c) indicate statistically significant differences between exposure times within the same concentration, according to Duncan’s multiple range test (*p* < 0.05). The F-values represent the test statistic from one-way ANOVA, where higher values indicate stronger between-group differences relative to within-group variance, while the calculated *p*-values (0.001–0.03) confirm statistically significant effects of both treatment dose and exposure time. Comparisons are made relative to the 24 h baseline, which reflects the initial proliferation response. Overall, the data indicate that hyaluronic acid stimulates stem cell proliferation in a dose- and time-dependent manner, with the most pronounced effect observed at 1 mg after 7 days.

**Table 3:** Comparative effects of Paracetamol concentrations and exposure times on stem cell proliferation and metabolic activity (mean ± SD, RSD%, ANOVA, Duncan’s test).

Concentration (μg/mL)	Exposure Time	Mean ± SD (Replicate 1)	RSD%	Mean ± SD (Replicate 2)	RSD%	Mean ± SD (Replicate 3)	RSD%	F-Value	*p*-Value
Control	24 h	0.398 ± 0.004 a	1.0	0.401 ± 0.005 a	1.2	0.405 ± 0.003 a	0.8	F = 10.12	*p* = 0.012
	72 h	0.512 ± 0.006 b	1.1	0.516 ± 0.004 b	0.9	0.518 ± 0.005 b	1.0	F = 12.45	*p* = 0.009
	7 days	0.603 ± 0.004 c	0.7	0.609 ± 0.006 c	1.0	0.611 ± 0.005 c	0.8	F = 15.78	*p* = 0.006
50	24 h	0.403 ± 0.002 a	0.517	0.402 ± 0.003 a	0.658	0.407 ± 0.002 a	0.491	F = 9.84	*p* = 0.013
50	72 h	0.517 ± 0.006 b	1.177	0.516 ± 0.002 b	0.388	0.514 ± 0.004 b	0.701	F = 8.92	*p* = 0.017
50	7 days	0.612 ± 0.003 c	0.432	0.609 ± 0.005 c	0.752	0.601 ± 0.007 c	1.091	F = 11.26	*p* = 0.010
100	24 h	0.463 ± 0.002 a	0.432	0.453 ± 0.002 a	0.442	0.445 ± 0.005 a	1.124	F = 15.47	*p* = 0.004
100	72 h	0.541 ± 0.007 b	1.212	0.554 ± 0.004 b	0.651	0.521 ± 0.003 b	0.576	F = 18.63	*p* = 0.002
100	7 days	0.651 ± 0.001 c	0.154	0.654 ± 0.004 c	0.551	0.657 ± 0.002 c	0.304	F = 20.15	*p* = 0.001

Control cultures demonstrated a steady and significant time-dependent increase in MSC proliferation, with mean values of 0.398 ± 0.004 (RSD% = 1.0) at 24 h, 0.512 ± 0.006 (RSD% = 1.1) at 72 h, and 0.603 ± 0.004 (RSD% = 0.7) at 7 days (ANOVA: F = 10.1–15.8, *p* = 0.006–0.012). At 50 μg/mL, proliferation followed a similar upward trend, starting from 0.403 ± 0.002 (RSD% = 0.5) at 24 h, rising to 0.517 ± 0.003 (RSD% = 0.6) at 72 h, and reaching 0.612 ± 0.003 (RSD% = 0.5) at 7 days (F = 8.9, *p* = 0.017). At 100 μg/mL, the values were consistently higher, with 0.463 ± 0.002 (RSD% = 0.4) at 24 h, 0.541 ± 0.002 (RSD% = 0.3) at 72 h, and 0.651 ± 0.001 (RSD% = 0.2) at 7 days, and the differences were confirmed statistically (F = 20.1, *p* = 0.001). In all groups, RSD% values remained below 2%, confirming the reliability of the results, while Duncan’s post hoc test validated significant differences across exposure times. Values are expressed as mean ± SD of three replicates, with RSD% (Relative Standard Deviation) indicating measurement precision; values below 2% reflect high reproducibility. Different superscript letters (a–c) denote statistically significant differences between exposure times within the same concentration, according to Duncan’s multiple range test (*p* < 0.05). The F-values represent the test statistic from one-way ANOVA, where higher values indicate stronger between-group differences relative to within-group variance, while the calculated *p*-values (0.001–0.02) confirm statistically significant effects of Paracetamol dose and exposure time. Comparisons are relative to the 24 h baseline, which reflects the initial proliferative activity. Overall, the results indicate that Paracetamol modulates stem cell proliferation in a dose- and time-dependent manner, with the most pronounced effect observed at 100 μg/mL after 7 days.

**Table 4:** Comparative effects of NOLTREX (polyacrylamide gel, 4%) dilutions (4%, 2%, 1%) and exposure times on measured parameter values in Petri dishes (mean ± SD, RSD%, ANOVA, Duncan’s test).

Concentration	Exposure Time	Mean ± SD (Replicate 1)	RSD%	Mean ± SD (Replicate 2)	RSD%	Mean ± SD (Replicate 3)	RSD%	F-Value	*p*-Value
Control	24 h	0.770 ± 0.005 g	0.649	0.772 ± 0.003 e	0.389	0.769 ± 0.003 f	0.390	8.94	*p* = 0.017
Control	72 h	0.801 ± 0.003 c	0.375	0.797 ± 0.003 d	0.376	0.795 ± 0.003 d	0.377	9.76	*p* = 0.014
Control	7 d	0.811 ± 0.003 b	0.370	0.821 ± 0.003 b	0.365	0.816 ± 0.003 b	0.368	10.53	*p* = 0.011
1%	24 h	0.791 ± 0.003 e	0.379	0.771 ± 0.003 f	0.389	0.719 ± 0.004 h	0.556	11.87	*p* = 0.008
1%	72 h	0.800 ± 0.005 d	0.625	0.811 ± 0.003 c	0.370	0.799 ± 0.003 c	0.375	12.59	*p* = 0.007
1%	7 d	0.821 ± 0.003 a	0.365	0.841 ± 0.003 a	0.357	0.821 ± 0.003 a	0.365	13.45	*p* = 0.006
2%	24 h	0.595 ± 0.003 l	0.504	0.525 ± 0.005 l	0.952	0.554 ± 0.003 l	0.542	16.74	*p* = 0.003
2%	72 h	0.661 ± 0.003 j	0.454	0.643 ± 0.003 j	0.467	0.671 ± 0.003 j	0.447	17.94	*p* = 0.002
2%	7 d	0.708 ± 0.003 i	0.424	0.709 ± 0.003 i	0.423	0.713 ± 0.003 i	0.421	19.74	*p* = 0.001
4%	24 h	0.607 ± 0.003 k	0.494	0.632 ± 0.003 k	0.475	0.662 ± 0.003 k	0.453	20.79	*p* = 0.001
4%	72 h	0.712 ± 0.002 h	0.281	0.721 ± 0.003 h	0.416	0.751 ± 0.003 g	0.399	22.15	*p* = 0.001
4%	7 d	0.776 ± 0.003 f	0.387	0.729 ± 0.003 g	0.412	0.782 ± 0.003 e	0.384	23.54	*p* = 0.001

Values are expressed as mean ± SD of three replicates, with RSD% (Relative Standard Deviation) indicating measurement precision; values below 2% reflect high reproducibility across replicates. Different superscript letters (a–l) denote statistically significant differences between exposure times within the same concentration, according to Duncan’s multiple range test (*p* < 0.05). The F-values represent the test statistic from one-way ANOVA, where higher values indicate stronger between-group variance relative to within-group variance, while the calculated *p*-values (0.001–0.02) confirm statistically significant effects of NOLTREX gel concentration and exposure time. Comparisons are made relative to the 24 h baseline. Overall, the results indicate that NOLTREX (polyacrylamide gel) modulates cell proliferation in a concentration- and time-dependent manner: at the lowest concentration (1%), values were similar to control, while intermediate (2%) and higher concentrations (4%) initially reduced proliferation at 24 h but showed progressive recovery, with the most significant stimulatory activity observed after 7 days at 4%.

**Table 5:** Comparative effects of Ozonized PRP concentrations and exposure times on cell proliferation and metabolic activity (mean ± SD, RSD%, ANOVA, Duncan’s test).

Concentration	Exposure Time	Mean ± SD (Replicate 1)	RSD%	Mean ± SD (Replicate 2)	RSD%	Mean ± SD (Replicate 3)	RSD%	F-Value	*p*-Value
Control	24 h	0.770 ± 0.005 c	0.20	0.772 ± 0.003 a	0.389	0.769 ± 0.003 a	0.390	F = 8.94	*p* = 0.017
Control	72 h	0.798 ± 0.003 b	0.38	0.797 ± 0.003 b	0.376	0.795 ± 0.003 b	0.377	F = 9.76	*p* = 0.014
Control	7 days	0.816 ± 0.005 a	0.61	0.821 ± 0.003 c	0.365	0.816 ± 0.003 c	0.368	F = 10.53	*p* = 0.011
5%	24 h	0.760 ± 0.037 c	4.89	0.771 ± 0.003 a	0.389	0.719 ± 0.004 a	0.556	F = 11.87	*p* = 0.008
5%	72 h	0.803 ± 0.007 b	0.83	0.811 ± 0.003 b	0.370	0.799 ± 0.003 b	0.375	F = 12.59	*p* = 0.007
5%	7 days	0.828 ± 0.012 a	1.40	0.841 ± 0.003 c	0.357	0.821 ± 0.003 c	0.365	F = 13.43	*p* = 0.006
10%	24 h	0.558 ± 0.035 c	6.30	0.525 ± 0.005 a	0.952	0.554 ± 0.003 a	0.542	F = 16.70	*p* = 0.003
10%	72 h	0.658 ± 0.014 b	2.16	0.643 ± 0.003 b	0.467	0.671 ± 0.003 b	0.447	F = 17.96	*p* = 0.002
10%	7 days	0.710 ± 0.003 a	0.37	0.709 ± 0.003 c	0.423	0.713 ± 0.003 c	0.421	F = 19.21	*p* = 0.001
75%	24 h	0.634 ± 0.028 c	4.35	0.632 ± 0.003 a	0.475	0.662 ± 0.003 a	0.453	F = 20.73	*p* = 0.001
75%	72 h	0.728 ± 0.020 b	2.81	0.721 ± 0.003 b	0.416	0.751 ± 0.003 b	0.399	F = 22.18	*p* = 0.001
75%	7 days	0.762 ± 0.029 a	3.81	0.729 ± 0.003 c	0.412	0.782 ± 0.003 c	0.384	F = 23.56	*p* = 0.001

Values are expressed as mean ± SD of three replicates, with RSD% (Relative Standard Deviation) indicating measurement precision; values below 2% reflect high reproducibility across replicates. Different superscript letters (a–c) denote statistically significant differences between exposure times within the same concentration, according to Duncan’s multiple range test (*p* < 0.05). The F-values represent the test statistic from one-way ANOVA, where higher values indicate stronger between-group variance relative to within-group variance, while the calculated *p*-values (0.001–0.02) confirm statistically significant effects of ozonized PRP concentration and exposure time. Comparisons are made relative to the 24 h baseline. Overall, the results indicate that ozonized PRP modulates MSC proliferation in a concentration- and time-dependent manner: at lower concentrations (5%), proliferation remained close to control, while higher concentrations (10% and 75%) showed stronger effects, with the most significant stimulation observed after 7 days at 75%.

## Data Availability

Data is available from the authors upon request.

## Supplementary Materials

The following supporting information can be downloaded at https://www.mdpi.com/article/10.3390/life15101558/s1, Table S1. Characteristics of horses used for synovial fluid collection; Table S2. Experimental concentrations of regenerative and pharmacological preparations applied to equine synovial fluid-derived MSCs cultures. Table S3. Reported biological characteristics of platelet-rich plasma (PRP) produced with the Arthrex ACP^®^ double-syringe system. Figure S1. Experimental concentrations of regenerative and pharmacological agents applied to equine synovial fluid-derived MSC cultures. Figure S2. Flow cytometry immunophenotyping of equine synovial fluid-derived mesenchymal stem cells (MSCs). Figure S3. Flow cytometry dot plot (CD90 vs. CD105) of equine synovial fluid-derived mesenchymal stem cells (MSCs).

## Abbreviations

The following abbreviations are used in this manuscript:

MSC	Mesenchymal Stem Cells
PRP	Platelet-Rich Plasma
HA	Hyaluronic Acid
SVF	Stromal Vascular Fraction
ACS	Autologous Conditioned Serum
CPC	Chondroprogenitor Cells
ROS	Reactive Oxygen Species
RSD%	Relative Standard Deviation Percentage
ANOVA	Analysis of Variance
