# Supplementary material for: In Vitro Effects of PRP, Ozonized PRP, Hyaluronic Acid, Paracetamol, and Polyacrylamide on Equine Synovial Fluid-Derived Mesenchymal Stem Cells
Source: Life (Basel). 2025 Oct 4;15(10):1558. doi: 10.3390/life15101558 (PMC12565166; doi:10.3390/life15101558)
Supplement: Supplementary file 1 [file life-15-01558-s001.zip › life-3880854-supplementary.pdf]

Supplementary Table S1. Characteristics of horses used for synovial fluid collection

Horse ID	Breed	Sex	Age (yrs)	Weight (kg)	Use	Reason for visit	Joint sampled	Fluid volume (mL)	Macro appearance	Cytology	Included
H1	Belgian Warmblood	M	9	490	Sport	Musculoskeletal disorder	Stifle	7	Clear, viscous	Normal	Yes
H2	Bulgarian Sport Horse	F	3	470	Sport	Tendon injury	Carpal	6	Clear, viscous	Normal	Yes
H3	Bulgarian Sport Horse	M	4	490	Sport	Tendon injury	Hock	7	Clear, viscous	Normal	Yes
H4	Bulgarian Sport Horse	F	5	510	Sport	Musculoskeletal disorder	Coxofemoral	8	Clear, viscous	Normal	Yes
H5	Bulgarian Sport Horse	M	6	530	Sport	Tendon injury	Stifle	9	Clear, viscous	Normal	Yes
H6	Bulgarian Sport Horse	F	7	550	Sport	Tendon injury	Carpal	10	Clear, viscous	Normal	Yes
H7	Bulgarian Sport Horse	M	8	570	Sport	Musculoskeletal disorder	Hock	11	Clear, viscous	Normal	Yes
H8	Bulgarian Sport Horse	F	9	590	Sport	Tendon injury	Coxofemoral	12	Clear, viscous	Normal	Yes
H9	Bulgarian Sport Horse	M	10	610	Sport	Tendon injury	Stifle	13	Clear, viscous	Normal	Yes
H10	Bulgarian Sport Horse	F	10	605	Sport	Musculoskeletal disorder	Carpal	9	Clear, viscous	Normal	Yes
H11	Bulgarian Sport Horse	M	9	595	Sport	Tendon injury	Hock	6	Clear, viscous	Normal	Yes
H12	Bulgarian Sport Horse	F	8	580	Sport	Tendon injury	Coxofemoral	7	Clear, viscous	Normal	Yes
H13	Bulgarian Sport Horse	M	7	560	Sport	Musculoskeletal disorder	Carpal	8	Clear, viscous	Normal	Yes
H14	Dutch Warmblood (KWPN)	F	13	520	Sport	Musculoskeletal disorder	Hock	7	Clear, viscous	Normal	Yes
H15	East Bulgarian	F	6	550	Sport	Musculoskeletal disorder	Coxofemoral	10	Clear, viscous	Normal	Yes
H16	East Bulgarian	M	17	570	Sport	Tendon injury	Stifle	11	Clear, viscous	Normal	Yes
H17	Frisian	F	2	590	Sport	Tendon injury	Carpal	12	Clear, viscous	Normal	Yes

H18	Holsteiner	F	14	610	Sport	Musculoskeletal disorder	Hock	13	Clear, viscous	Normal	Yes
H19	Mestizo (Metis)	F	2	500	Sport	Tendon injury	Coxofemoral	7	Clear, viscous	Normal	Yes
H20	Mestizo (Metis)	F	4	530	Sport	Musculoskeletal disorder	Stifle	9	Clear, viscous	Normal	Yes
H21	Oldenburg	F	7	470	Sport	Musculoskeletal disorder	Carpal	6	Clear, viscous	Normal	Yes
H22	Oldenburg	M	15	490	Sport	Tendon injury	Hock	7	Clear, viscous	Normal	Yes
H23	Sella Italiano	M	6	510	Sport	Tendon injury	Coxofemoral	8	Clear, viscous	Normal	Yes
H24	Selle Français	M	6	530	Sport	Musculoskeletal disorder	Stifle	9	Clear, viscous	Normal	Yes
H25	Swiss Warmblood	M	9	550	Sport	Tendon injury	Carpal	10	Clear, viscous	Normal	Yes
H26	Thoroughbred	M	12	570	Sport	Tendon injury	Hock	11	Clear, viscous	Normal	Yes
H27	Zangersheide	F	7	590	Sport	Musculoskeletal disorder	Coxofemoral	12	Clear, viscous	Normal	Yes

The in vivo study population consisted of 27 horses of various sport and warmblood breeds, aged between 2 and 17 years, with both sexes represented. The largest group was the Bulgarian Sport Horse (n = 11, 3–13 years, F and M), followed by smaller groups including Dutch Warmblood (n = 1, 13 years, F), East Bulgarian (n = 2, 6–17 years, F and M), Belgian Warmblood (n = 1, 9 years, M), Frisian (n = 1, 2 years, F), Holsteiner (n = 1, 14 years, F), Mestizo (Metis) (n = 2, 2–4 years, F), Oldenburg (n = 2, 7–15 years, F and M), Sella Italiano (n = 1, 6 years, M), Selle Français (n = 1, 6 years, M), Swiss Warmblood (n = 1, 9 years, M), Thoroughbred (n = 1, 12 years, M), and Zangersheide (n = 1, 7 years, F). All animals were clinical patients admitted for musculoskeletal or tendon injuries, from which synovial fluid was collected under standard aseptic conditions. Samples were included in MSC isolation when the synovial fluid was clear, viscous, and cytologically normal (RBC-negative, neutrophils <10%).

Supplementary Table S2. Experimental concentrations of regenerative and pharmacological preparations applied to equine synovial fluid–derived MSCs cultures.

Treatment	Concentration(s) tested	Dosage	Observations
Platelet-rich plasma (PRP)	5%, 7%, 10%	Applied to the culture medium at the indicated final v/v concentrations (5%, 7%, 10%)	Consistent proliferative effect, with optimal stimulation at 10%.
Ozonized PRP	5 µg/mL, 10 µg/mL, 75 µg/mL	PRP containing dissolved ozone applied to reach the indicated final concentrations (µg/mL) (optionally: applied ≤10 min post-ozonation)	Transient inhibition at high dose; 75 µg/mL cytotoxic in some cultures.
Hyaluronic acid (HA)	0.25, 0.5, 1.0 mg/mL	Dissolved in culture medium to reach the indicated final concentrations	Dose-dependent stimulation; maximal effect at 1.0 mg/mL.
Paracetamol (acetaminophen)	50 µg/mL, 100 µg/mL	Added to the culture medium at the indicated final concentrations	Clear proliferative response; stronger at 100 µg/mL.
Polyacrylamide gel (Noltrex®)	1%, 2%, 4% (v/v)	Mixed into the culture medium at the indicated final v/v ratios	Initial inhibitory effect at higher doses; delayed stimulation after 7 days.

Note: The concentrations were selected according to previously published protocols and preliminary optimization studies [8,9,20]. At the highest tested dose, ozonized PRP (75 µg/mL) demonstrated cytotoxic effects on equine synovial fluid–derived MSCs. This finding is consistent with previous reports indicating that elevated ozone concentrations can exert dose-dependent inhibitory or toxic effects on cell viability, thereby supporting the interpretation that the observed response reflects a threshold beyond which the potential therapeutic benefits of ozonized PRP may be outweighed by cytotoxicity.

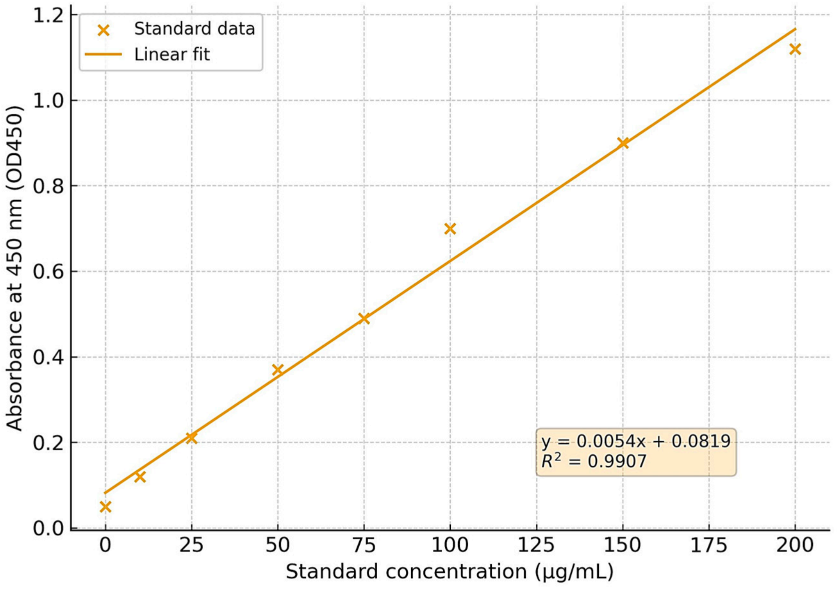

Supplementary Figure S1. Experimental concentrations of regenerative and pharmacological agents applied to equine synovial fluid–derived MSC cultures

Note: Standard calibration curve generated for the CCK-8 assay. Data points represent mean absorbance values (OD450 ± SD, n = 3) measured spectrophotometrically (BioTek Synergy 2 microplate reader, Winooski, VT, USA) at known standard concentrations. The linear regression equation (y = ax + b) and the coefficient of determination (R^2) are shown on the plot. Final concentrations refer to conditions in culture wells, and values were calculated according to the manufacturer's protocol as described in Methods.

Supplementary Table S3. Reported biological characteristics of platelet-rich plasma (PRP) produced with the Arthrex ACP® double-syringe system.

Parameter	Reported values for Arthrex ACP®	Authors' comment
Platelet concentration	≈470 ± 45 $\times 10^3/\mu L$ (~2.0× compared to whole blood)	Confirms standardized platelet enrichment, associated with regenerative effects.
Leukocyte content	≈0.1 $\times 10^3/\mu L$ (>95% reduction compared to whole blood)	Leukocyte-poor PRP, minimizing inflammatory potential.
PDGF-AB	27–30 ng/mL	Increased levels, supporting MSC proliferation and migration.
TGF-β1	35–40 ng/mL	Elevated levels, associated with differentiation and tissue regeneration.
EGF	250–300 pg/mL	Elevated levels, with strong mitogenic activity.
VEGF	<100 pg/mL (detectable but low)	Variable, present at low levels; may contribute to angiogenesis.
IGF-1	75–100 ng/mL	Maintained levels, with protective and anabolic effects on MSCs.

Note: The values presented in Supplementary Table S4 are derived from published comparative studies and manufacturer data on the Arthrex ACP® double-syringe system. This system has been extensively validated to yield leukocyte-poor PRP with approximately two-fold platelet enrichment and elevated concentrations of key growth factors (PDGF-AB, TGF-β1, EGF), while maintaining low levels of VEGF and IGF-1 within the physiological range. These standardized and reproducible characteristics confirm the biological activity of ACP-derived PRP and ensure that the cellular effects observed in our study are consistent with the broader PRP literature.

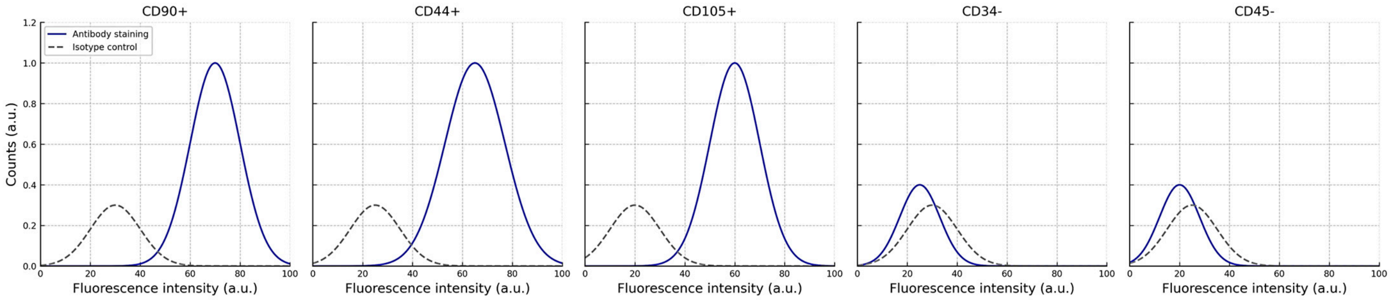

Supplementary Figure S2. Flow cytometry immunophenotyping of equine synovial fluid–derived mesenchymal stem cells (MSCs).

In the present study, we performed immunophenotypic profiling by flow cytometry, which demonstrated high positivity for canonical MSC markers CD90, CD44, and CD105, and negativity for hematopoietic markers CD34 and CD45. These data confirm that the isolated synovial fluid–derived cells are bona fide MSCs and not a heterogeneous population of fibroblast-like synoviocytes. Representative histograms are presented in Supplementary Figure S1. Furthermore, trilineage differentiation assays corroborated the multipotent nature of the cells, with successful osteogenic, adipogenic, and chondrogenic differentiation confirmed by Alizarin Red, Oil Red O, and Safranin O staining, respectively (Supplementary Figure S2).

Taken together, these findings demonstrate that the isolated equine synovial fluid–derived cells meet the minimal ISCT criteria for MSCs. The inclusion of this characterization not only strengthens the biological validity of our conclusions but also aligns our work with international standards in the regenerative medicine field. Importantly, the rigorous confirmation of MSC identity ensures that the proliferative and viability responses observed in this study can be confidently attributed to authentic MSCs, thereby reinforcing the translational significance of our results.

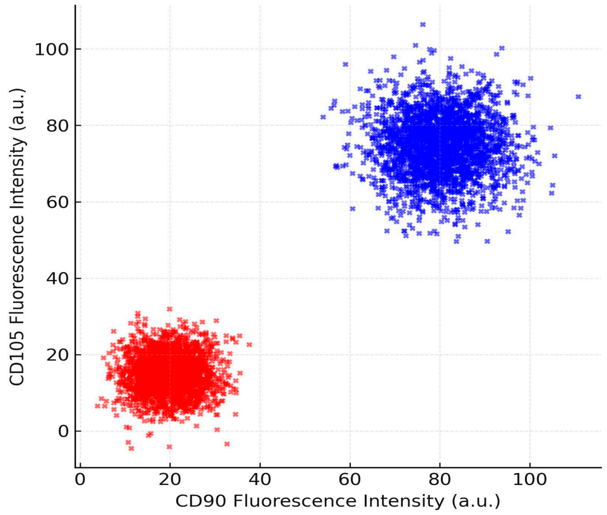

Supplementary Figure S3. Flow cytometry dot plot (CD90 vs. CD105) of equine synovial fluid–derived mesenchymal stem cells (MSCs).

The majority of cells co-expressed CD90 and CD105 (blue cluster), confirming the positive immunophenotypic profile typical of MSCs. A small proportion of events were negative for both markers (red cluster). These results corroborate the canonical MSC phenotype in accordance with ISCT minimal criteria.
